# Enhanced “Green Nudging”: Tapping the channels of cultural transmission

**DOI:** 10.1371/journal.pone.0328434

**Published:** 2025-08-08

**Authors:** Christian Cordes, Joshua Henkel

**Affiliations:** University of Bremen, Bremen, Germany; Otago Polytechnic, NEW ZEALAND

## Abstract

This paper shows how to increase the effectiveness of “green nudging” as a policy measure to induce sustainable preferences. Evidence indicates that the behavioral impact of “green nudges” is subject to decay. To address this problem, we propose “enhanced green nudges”, which incorporate learning biases as features of humans’ capacity for culture. These provide information for the formation of enduring “green” preferences. Several biases in cultural transmission are considered, such as direct bias, norm-following behavior, conformity, self-similarity, and the influence of role models. Moreover, a prerequisite for “enhanced green nudges” to be effective is that learning environments resemble the ones biases evolved from in humans’ evolutionary past. Based on a model of cultural evolution, several scenarios of preference acquisition through “green nudges” illustrate our arguments and indicate implications for policy.

## 1. Introduction

This article shows how the behavioral effectiveness of “green nudging” can be increased. It represents an important policy measure to promote sustainable preferences among consumers [1,2]. “Nudges” are defined as attempts to influence the choice of an agent in a predictable way, for example, by presenting a decision problem in a certain manner, without changing monetary incentives or the option set itself [3,4]. Evidence shows, however, that the impact of “green nudges” on agents’ behavior is rather short-lived and subject to decay [5–13]. We therefore propose “enhanced green nudges” that incorporate learning biases as features of humans’ capacity for culture. These learning dispositions then provide information for the enduring acquisition of “green” preferences. Several biases in cultural transmission are considered, such as direct bias, norm-following behavior, conformity, self-similarity, and the influence of role models. Moreover, we claim that a prerequisite for ”enhanced green nudges” to be effective are learning environments similar to those biases evolved from in humans’ evolutionary past [14–17].

Cultural transmission of information influenced by these biases determines a great share of individuals’ sets of preferences that are shaped by learning in social environments [17,18]. Human preferences are, therefore, neither fixed nor homogenous, but malleable [19–22]. Agents individually and socially learn new ones or modify existing ones [23–25]. We suggest that learning biases in cultural transmission can be harnessed by “enhanced green nudges” to shift agents toward environmentally benign preferences. In this context, we define “enhanced green nudges” as instruments that provide information subject to learning biases for the formation of enduring “green” preferences. Furthermore, when operating in environments similar to those they evolved in, these learning forces are strong drivers of behavioral adaptation and preference change [15,26,27].

Below, we propose a model of cultural evolution that incorporates recursion equations capturing several forces of preference acquisition. It enables the systematic analysis of the interplay of “green nudging”, its fading phenomena, preference acquisition via “enhanced green nudges”, individual learning, and the inducement of preferences via defaults (the formal setup draws on [14,28–30]). We derive implications concerning the effectiveness of “enhanced green nudges” in population-based preference learning. The model also accounts for social environments that vary as to the extent to which their characteristics affect the strength of cognitive biases in cultural transmission. Several scenarios based on different parameter constellations, which are informed by empirical evidence or used to categorically discriminate strong and weak effects of particular learning forces, illustrate our arguments. We differentiate the long-term effectiveness of “green nudges” by conceptually discriminating the dissemination of “green” preferences from the level of overt “green” behaviors in a population.

Based on these scenarios, we propose that features of cultural transmission can serve as ingredients to powerful political instruments. “Enhanced green nudges” affect an individual’s choice by providing information through biased learning. For instance, a political initiative invoking a role model bias as well as self-similarity as components of an “enhanced green nudge” promote “green choice”: organized direct communication between acknowledged “green” experts and pioneer adopters can induce the latter’s acquisition of a “green” preference. As models that enjoy prestige among peers, these early adopters introduce the “green” choice to their local group of self-similar peers by means of face-to-face contacts and assignability, as characteristics of a learning environment suited for “enhanced green nudges”. After having convinced a critical number of adopters in a community, diffusion is supported by conformity – another “nudge” influencing an agent’s choice and potential tool for policy. In addition, subsidies promoting the initial adoption of “green” behaviors increase their visibility in local groups’ learning environment and suggest a state-backed norm. Politicians can then harness norm psychology as a “nudge” to foster its dissemination. Moreover, “green” status races fueled by direct bias in cultural transmission could be tapped as a nudge to promote “green” choice. Below, we show how orchestrated sequences of “enhanced green nudges” in certain learning settings can be implemented to spread “green” behavior.

Findings on cognitive biases in individuals’ acquisition of information for preference formation led to the emergence of research fields such as “behavioral economics” [31] and “behavioral environmental economics” [4]. Mostly, these avenues interpret biases as systematic deviations from rationality. Their insights have been used to modify agents’ choice architectures to direct, or “nudge”, decisions toward ecologically more sustainable behavior [1,3,32–36]. However, biases have also been studied by anthropology in strictly positive terms, without normative connotations [27]. These approaches start from the observation that humans routinely face complex environments in which they have to deal with cognitive constraints on the processing of information. Confronted with these settings in its evolutionary past, the human brain evolved specialized psychological skills to acquire, store, process, and organize the knowledge available in individuals’ cultural environments [16,27,30,37–39]. The coevolutionary process between genes and culture created traits of humans’ psyche highly relevant in explaining contemporary behavior.

The paper is organized as follows. Section 2 discusses the role of “nudges” in attempts to influence agents’ consumption choices and substantiates the notion of “enhanced green nudges”. Next, Section 3 proposes a model of cultural evolution that integrates our arguments in a formal structure. Based on this model, Section 4 presents several scenarios of population-based preference acquisition in different learning environments. Some propositions are offered and implications for environmental policy are derived. Section 5 concludes.

## 2. From “Green Nudges” to “Enhanced Green Nudges”

“Nudges” are established instruments in environmental policy that modify choice architectures to induce behavioral adjustments. These “soft” interventions into decision-making are legitimated by societal goals, for example, the transition toward environmentally sustainable preferences [1,8,13]. “Nudges” have several advantages over traditional regulation: they are cost effective as compared to laws or marketing campaigns, are easy to implement, and are often widely accepted by the population [2,40]. The most frequent critique of “nudges” addresses their paternalistic and manipulative character. Thaler and Sunstein [41], therefore, recommend a high level of transparency when introducing “nudges” and suggest a “libertarian paternalism” that, while modifying choice architectures, respects individuals’ general freedom of choice.

An important question is whether “green nudges” promote long-run behavioral change or merely trigger short-term adjustments. In fact, most of them are limited as to long-term effects on agents’ behaviors [6–10,42,43]. Momsen and Stoerk [13] show that “nudges” including priming, mental accounting, framing, and decoy proved ineffective. Also mentioning social norms – without visibility of behavior or direct communication among agents – failed to lastingly induce environmentally benign behavior. Allcott and Rogers [12] observe that consumers’ energy saving efforts invoked by abstract social comparisons as a “green nudge” in home energy reports decay relatively quickly after “nudging” stops (also [11]). Evidence reviewed by Abrahamse and Steg [36] suggests that in before-after settings the “nudge”-based treatment effect dissipates when the measure is no longer in place. The initial effect only becomes more persistent (albeit at low levels) if the intervention continues over longer periods of time. Consequently, a shortcoming concerns the long-term effect of “green nudges”: they exhibit behavioral effects subject to fading processes. The only class of “nudges” that entailed more persistent behavioral effects are default-based “nudges” [4]. Pichert and Katsikopoulos [44] show how a default alters energy consumption: citizens of Schönau in Germany, conservative voters by majority, stayed with the default option “green energy” set by the local utility even though opting-out was easy, the option opposed their political attitudes, and was more expensive (also [45]). A “status quo bias” that combines “loss aversion” and “endowment effects” can explain this behavior: agents value the preset option relatively higher because it is regarded as being integral part of their endowment and thus serves as a reference point. Opting out of the default then implies a loss [46]. We propose that the “status quo bias” draws on evolved psychological dispositions, which makes it part of the category of “enhanced green nudges”. Our model of cultural evolution introduces an “inducement effect” in preference acquisition emanating from default-based “nudges”.

Other approaches to “nudging” either support our notions or focus on particular features that deviate from our understanding. Starting from the observation that behavior reverts to the preintervention state when the “nudge” has been removed, Hertwig and Grüne-Yanoff [47] suggest “boosts”, i.e., the development of agents’ competences to make their own choices, to cause persistent behavioral effects. This is compatible with our concept, since besides providing information for preference acquisition, cultural transmission is also suited to pass on various skills to other agents [48,49]. Löfgren and Nordblom [50] suggest that agents are “nudgeable” in situations of “inattentive choice” only. While the provision of information through “enhanced green nudges” may be unconscious, resulting preference formation based on this information may still enter deliberate reasoning. Supporting our view, Banerjee and John [51] argue that a “nudge plus” becomes more effective and entails sustainable change in behavior if it necessitates reflection and deliberation. Researchers in cognitive science suggest social learning strategies and norm-following behavior to rest on domain-general psychological processes combined with culturally evolved, domain-specific “cognitive gadgets” adopted early in life [52,53]. This idea is not at odds with our concept of “enhanced green nudging” that may also incorporate “cognitive gadgets”. Moreover, a “cognitive gadget” acquired from an individual’s environment during childhood may function best in settings that resemble small-group socialization contexts including family, friends, close peers, conformity and role models.

Throughout an individual’s life, her malleable preferences are subject to individual and social learning processes [24]. Since humans are cultural beings, biased cultural transmission takes center stage in this context [16,49]. In suited learning environments, “enhanced green nudges” incorporating features of humans’ capacity for culture have the potential to induce lasting shifts in preferences. We focus on the following cognitive biases affecting information provision through cultural transmission as components of “enhanced green nudges”:

(1) Preference acquisition features direct biases [27,29]. Due to evolved cognitive dispositions that increase the inherent attractiveness of particular cultural traits, directly-biased people preferentially adopt these traits based on their contents [16,54,55]. A case in point are preferences for goods or behaviors suited to signal status due to inherent characteristics, such as exclusiveness [56–58]. People care about their relative position in society. This deeply-rooted disposition entails an inherent attractiveness of status-signaling goods and a direct bias favoring them. Due to “rat race” dynamics in status-based consumption, it tends to be resource-intensive. However, also environmentally benign behaviors can serve as status-signaling means in communities indicating, for example, avant-garde behavior. Status-driven consumption would then foster the dissemination of “green” consumption. Hence, direct biases may be components of “enhanced green nudges”. Moreover, behaviors that yield agents superior hedonistic experiences can give rise to direct bias in individual learning.
(2) A strong evolved bias that takes effect in cultural transmission is conformity. It represents a heuristic to figure out well-adapted kinds of behavior by drawing on the frequency with which they are shown by agents in a particular cultural context [59–62]. Agents are more likely to pick the behavioral variant that is modeled by the majority of group members, i.e., they discriminate against behaviors that are rare among peers. Positive and normative conformity explain many aspects of group behavior including nonlinear behavioral changes, such as threshold and critical mass phenomena [63]. Once a “green” behavior is displayed by the majority in a peer group, this bias fosters its spreading. On the other hand, it may also favor environmentally harmful behaviors prevalent in a population. In that case, other instruments have to be applied to lead the population out of this “lock-in” [32]. Conformity represents a central component of “enhanced green nudges”.
(3) Moreover, norm psychology as a disposition to comply with groups’ rules plays an important role in biased social learning and informs the implementation of “enhanced green nudges”. This disposition has been caused at the genetic level by the enforcement of social norms in group culture contexts [16,38,64]. Phenotypes endowed with a propensity to obey norms enjoyed a relative advantage in these settings. This psychological disposition was of adaptive value for it enabled the stabilization of cooperation and other prosocial norms at the group level [65]. Norm-following behavior supports environmentally benign behavior once it is established as a norm in a reference group, for example, via a prestigious role model [66]. Labels on environmentally friendly products not only provide additional information, they also allow consumers to reveal their norm-following lifestyle to the reference group [67,68].
(4) Finally, a particularly powerful force in cultural transmission and “enhanced green nudging” is the role-model bias. Evidence from psychology and anthropology shows that the adoption of behavioral traits is frequently conditioned by observable attributes of individuals exhibiting the trait [69–72]. Individuals who are perceived as models are characterized by attributes such as success, credibility, competence, status, or prestige [51,73]. Copying behaviors of role models again represents a simple evolved heuristic that enables social learners to identify behaviors well-adapted to complex environments [16,74]. A model-based bias is considered to be an indirect one that results if social learners use the value of a second trait that characterizes a model (e.g., prestige) to determine the attractiveness of that individual as a model for the primary trait (e.g., consumption behavior). It is more effective if a model is similar to the target individual along dimensions of self-similarity, such as sex, culture, dialect, ethnicity, regional provenance, socio-demographics, or other measures of proximity [75,76].

Since these learning biases feature deep evolutionary roots in human phylogeny and gene-culture coevolution, they strongly influence cognition and determine which information in an individual’s cultural environment will be subject to deliberate processing [77,78]. These cognitive dispositions crucially affect the constitution of an agent’s set of preferences that is, to a great extent, determined by cultural transmission forces. Incorporating social learning biases into “enhanced green nudges” turns these into powerful instruments in lastingly changing consumers’ preferences – a prerequisite for a transition toward “green” behaviors beyond mere temporary behavioral adaptations. However, an indispensable requirement for this behavioral impact to realize is that social learning takes place in “natural” settings including direct observation of (self-similar) models, face-to-face communication with peers in small group-contexts, or – with a lower effect – the presentation of norms via the media. We claim that the effectiveness of “enhanced green nudges” in cultural transmission hinges on whether the learning environment resembles the one biases originated from in our evolutionary past. Section 4 offers a more detailed discussion of these aspects of “enhanced green nudging” in the context of several scenarios based on a model of cultural evolution.

## 3. A model of “Green” preference acquisition

Our model of cultural evolution captures cultural transmission and individual learning underlying the acquisition of preferences for two behaviors, A and B. Let a be the preference for the “green” behavior,A. b denotes the one for the environmentally more harmful behavior B. Preferences represent a positive attitude toward the respective behavior and willingness to adopt it. The state of the population of N individuals is given by the frequency of agents that prefer A, i.e., those holding preference a labeled p (0≤p≤1). The share of b is given by 1−p. G measures the frequency of overt “green” behavior in the population 0≤G≤1, i.e., the observable consumption of A. As shown, the frequency of A among agents does not necessarily correspond with the grade of dissemination of the preference for it. A “green nudge” may introduce environmentally benign behavior to a population without changing people’s preferences. In that case, some of the “green” behavior originates from short-term behavioral adjustment. G is determined by the share of p and the application of “green nudges”. p is small in the beginning. We define recursion equations that predict the frequencies of p and G in the next stage of preference learning and “nudging”, $p^{\prime }$ and $G^{\prime }$, given their current frequencies. We include partial recursions capturing (1) the fading of “green nudges”, (2) the inducement of “green” preferences via a default, (3) direct bias in individual learning, and (4) cultural evolutionary forces that bias preference acquisition via social learning. We account for a model bias, conformity, self-similarity, and norm-following. (5) A dynamic system integrates these forces in preference acquisition.

### 3.1. Fading phenomena in “green nudging”

Observable “green” behavior introduced to a population by a “green nudge” is subject to decay. This is due to agents that, while displaying the “green” variant, do not acquire a stable preference for the environmentally benign option. Equation (1) formally describes this fading process: a recursion determines G in the next time step, $G^{\prime }$, given the value of G in this period and a fraction μG(G−p) of agents that switches from the “green” variant, A, to the conventional choice, B, thereby representing fading:


$$G^{\prime }=G-\mu G(G-p).$$ (1)

The equilibrium frequency of overt “green” behavior, $\hat{G}$, is calculated by subtracting G from both sides of $\;\left(G^{\prime }-G=0\right)$ and yields $\hat{G}=p$ or $\hat{G}=0$. If, after “green nudging” has been applied as a political instrument to the population, G>p, a fraction of the observed overt “green” behavior is not accompanied by a corresponding acquisition of a “green” preference. Behavioral change is merely temporarily induced by the “nudge”. As a result, G will decrease until G=p, i.e., we see a continuous decay of the “nudging” effect. The pace of fading hinges on the parameter μ that scales its strength. On the other hand, as long as p>G due to prior preference acquisition, the share of “green” behavior among agents, A, increases until the equilibrium at $\hat{G}=p$ is reached. Holding a “green” preference, a sooner or later translates into overt “green” behavior. This necessitates G≠0, i.e., at least one individual exhibiting A is required to trigger an increase of G.

### 3.2. The inducement of preferences via default-based “green nudges”

Despite the fact that also default-based “nudges” are subject to decay [12], this instrument potentially induces lasting shifts in agents’ preferences. Pichert and Katsikopoulos [44] show that a preset option affects preference acquisition: consumers asked for more money to give up the default “green electricity” than they were willing to pay for it. This relative effectiveness is, we argue, due to the phylogenetic roots of the underlying cognitive biases: the “endowment effect” and “loss aversion”. Due to that, they exert greater impact on agents’ reasoning. This inducement effect of “green” preferences by means of “default-based green nudging” is captured by a partial recursion that depicts the acquisition of a by a share of consumers:


$$p^{\prime }=p+\delta G(1-p).$$ (2)

In every time step, agents that have been subject to “default-nudging” (default set to A) measured by G , and that have not yet acquired a, captured by (1−p), potentially adopt the “green” preference. δ measures the strength of preference inducement by determining the share of G(1−p) that translates into p in the next stage. δ may vary across realms due to different affective evaluations of defaults: while pension schemes may be treated in a rather neutral way, this may not be the case in organ donation.

### 3.3. Directly biased preference learning

We extend Equation (2) with an expression for directly biased preference acquisition [14,32]. Direct biases originate from cognitive dispositions that increase the inherent attractiveness of a cultural trait [16,54,55]. For example, behaviors well-established to signal status to peers are favored in cultural transmission: they meet a basic need to define relative position in a group [56–58]. Direct bias may also emanate from individual learning: conventional commodities are often hedonistically more attractive in terms of sensory pleasures due to perfected performance, better supporting infrastructure, habits, or cost advantages [79,80] Therefore, in our model, a direct learning bias favors the acquisition of b. The “green” preferences may spread less or be crowded out by the conventional variant. To capture this superiority of B in preference learning, we assume that each a holder has a chance of learning to favor B, measured by $\gamma_{ab}$. A direct bias for “green” status goods would disseminate a. A fraction $\gamma_{ab}$ acquires preference b and is subtracted in the recursion, in each time step. Equation (2) modifies to


$$p^{\prime }=p+\delta G(1-p)-p\gamma_{ab}.$$ (3)

### 3.4. The acquisition of “green” preferences via social learning forces

The acquisition of “green” preferences is subject to consciously controlled processing of information. The provision of this information, in turn, strongly relies on cultural transmission [79,81,82]. As argued, cultural learning is biased: adopters tend to socially acquire some traits – in our case preferences – rather than others. To understand how cognition directs social learning, we account for these biases [79]: (1) the relevance of models in an agent’s social environment differs. Peer consumers in different social roles, with varying degrees of social proximity and diverse personal characteristics, the family, activist groups, science, experts, or the media vary in their influence. We assign different weights in cultural transmission to these entities, $\alpha_{i}$ ($\sum_{i}\alpha_{i}=1$). Models M2 and M3 represent peers whose influence in social learning is high due to their direct communication with the individual. They represent a “natural”, group-bound learning environment akin to those biases evolved in. These “live models” show either preference a or b, and may have changing weights ($\alpha_{2}$, $\alpha_{3}$). Less weight in cultural transmission is given to a dedicated “green” model M1 ($\alpha_{1}$) that always propagates a. M1 proxies the influence of more “abstract” models acting through “symbolic modeling” in the media or via publications. (2) To incorporate conformity into social learning, we look at sets of three models, whose frequency-dependent impact is expressed by parameter η.  0≤η≤1 implies that the weight of the more common preference among models is increased. If , conformity is absent. η=0Table 1 shows the probability of agents adopting preference a or b given a particular set of role models with different weights and conformity:

**Table 1:** Probability of acquiring preference a or b given a particular set of models (M1, M2, M3) with different weights ($\alpha_{1}$, $\alpha_{2}$, $\alpha_{3}$) and frequency-dependent transmission (η).

Preference of			Probability That Agent Acquires Preference	
M1	M2	M3	a	b
a	a	a	1	0
a	a	b	$\left(\alpha_{1}+\alpha_{2}\right)+\eta \left(1-\alpha_{1}-\alpha_{2}\right)$	$\alpha_{3}(1-\eta )$
a	b	a	$\left(\alpha_{1}+\alpha_{3}\right)+\eta \left(1-\alpha_{1}-\alpha_{3}\right)$	$\alpha_{2}(1-\eta )$
a	b	b	$\alpha_{1}(1-\eta )$	$\left(\alpha_{2}+\alpha_{3}\right)+\eta \left(1-\alpha_{2}-\alpha_{3}\right)$

From this table, the frequency of a after social learning, $p^{\prime \prime }$, given that it was $p^{\prime }$ before, is


$$\begin{array}{cc} p^{\prime \prime } & ={p^{\prime }}^{2}+p^{\prime }\left(1-p^{\prime }\right)\backslash \{\left(\alpha_{1}+\alpha_{2}\right)+\eta \left(1-\alpha_{1}-\alpha_{2}\right)+\left(\alpha_{1}+\alpha_{3}\right)+\eta \left(1-\alpha_{1}-\alpha_{3}\right)\backslash \}\\ & \;+{\left(1-p^{\prime }\right)}^{2}\left\{\alpha_{1}(1-\eta )\right\}\\ & =p^{\prime }-\left(p^{\prime }-1\right)\left(\alpha_{1}+\eta \left(p^{\prime }-\alpha_{1}\right)\right)\;, \end{array}$$ (4)

which constitutes the partial recursion for social learning including two cognitive biases. It computes the frequency of each set of models, multiplies this by the probability that a particular set results in an individual acquiring preference a, and then sums over all possible sets of models. M1 shows the green preference a with probability 1. The conformity parameter η favors the more common preference within the set of models. The recursion for p, obtained by substituting the partial recursions for default-based “green nudging” (2) and direct bias (3) into the recursion for social learning (4), is


$$p^{\prime \prime }=p+\delta G(1-p)-{p\gamma }_{ab}-\left(p+\delta G(1-p)-{p\gamma }_{ab}-1\right)\left(\alpha_{1}+\eta \left(p+\delta G(1-p)-p\gamma_{ab}-\alpha_{1}\right)\right).$$ (5)

### 3.5. A two-dimensional dynamic system for the acquisition of preferences

We yield two coupled recursions, one describing the development of the frequency of the “green” preference, a, in the population, denoted by p, (6) and another one for G (7), the overt “green” behavior among consumers (with $\Phi =\delta G(1-p)-{p\gamma }_{ab}$):


$$\Delta p=\Phi -(p+\Phi -1)\left(\alpha_{1}+\eta \left(p+\Phi -\alpha_{1}\right)\right)p^{\prime \prime }-p=\Delta p)$$ (6)

$$\Delta G=-\mu G(G-p)G^{\prime }-G=\Delta G).$$ (7)

In the context of several scenarios, we use this two-dimensional dynamic system to calculate our model’s equilibria for $\hat{p}$ and $\hat{G}$ (Δp=0 and ΔG=0).

## 4. Discussion: some scenarios

Several scenarios based on our model of cultural evolution show the interplay of behavioral decay, default-based “nudges”, individual and social learning dispositions, as well as a learning environment’s characteristics in preference acquisition. The model’s parameter values are either directly informed by existing empirical evidence, such as the fading rate of behavioral adjustments induced by “green nudges”, or set to categorically discriminate between strong and weak effects, such as in the case of a role model’s influence, conformity, or hedonistic individual learning. We find how “enhanced green nudges” incorporating a role model or conformity induce preference learning that counteracts behavioral decay. We also identify features of learning environments that affect the strength of cognitive biases in “enhanced green nudging”. These include typical aspects of small-group contexts, such as saliency and assignability of behavior, face-to-face interaction, norm-following, self-similarity, and local peers’ influence. We show how a default-based “nudge” serves to initiate “green” preference acquisition and observe an action-value-gap in agents’ behavior. Formally, by iterating the system for many individual and social learning as well as fading and inducement steps, we study its long-run properties and driving forces. The scenarios compare constellations of plausible model parameters and the resulting developmental paths for its key variables, the frequencies of the “green” preference, a, and the overt “green” behavior, G, among consumers. Moreover, they deliver several propositions as to the effects of “green nudges” on preference acquisition and suggest implications for environmental policy.

### 4.1. The decay of the behavioral effects of “green nudges”

**Fig 1:**
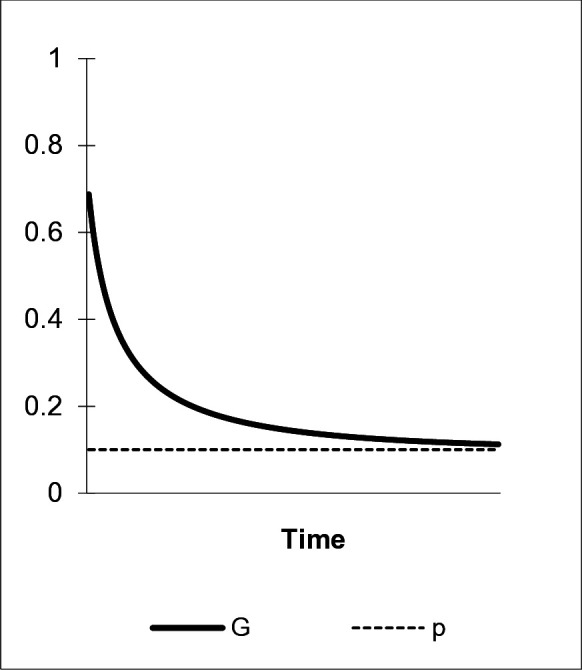
The fading effect. $\alpha_{1}=0$, η=0, $\gamma_{ab}=0$, μ=.2, δ=0.

Environmentally benign behavior introduced to a population by “green nudging” is subject to decay: as long as there is no change in underlying preferences, no motivation exists that permanently induces “green” choice. Shifts in behavior triggered by these interventions then exhibit a one-off effect as to behavioral adaptation. As a first scenario, (Fig 1) shows a stylized version of this process: the share of overt “green” behavior, G, is decreasing continuously in time, i.e., *ceteris paribus*, we observe a fading process after an initially high “post-nudge” level of environmentally benign behavior. The strength of the decay process is μ=.2, which is supported by empirical evidence [12,83]. G declines until its equilibrium frequency at $\hat{G}=p$ is reached. With $\alpha_{1}=0$, $\alpha_{2}=\alpha_{3}=\mathrm{.5}$ and η=0 social learning has no effect on the frequency of a. p represents an established share of agents who hold preference a and adopt the “green” behavior (p=.1). We suggest the following proposition:

**Proposition 1** *Overt environmentally benign behavior induced in a population by “green nudges” is subject to decay, if not accompanied by corresponding preference learning.*

### 4.2. “Enhanced green nudges” and learning environments

Central features of “natural” learning environments cognitive biases evolved from in humans’ phylogenetic past are face-to-face interactions with peers in small-group contexts [16,84]. Direct communication of this kind is an effective channel of cultural transmission to disseminate norms, beliefs, and knowledge, i.e., to provide the information for individuals’ deliberate change of preferences. Accordingly, direct social interaction has been shown empirically to be superior in spreading environmental knowledge and behavioral change: a review study by Abrahamse and Steg [36] concludes that face-to-face interaction is more effective in changing behavior than abstract feedback provision on the environmental behavior of other community members (also [76,85]). Young et al. [86] find that social media cannot replicate the influence of face-to-face interactions in reducing food waste. In the case of “green” electricity, personal communication with peers has been shown to be a crucial input to the deliberate adoption of corresponding “green” preferences [87,88]. Welsch and Kühling [81] suggest that direct communication in a community increases agents’ probability to subscribe to “green” electricity programs. They also attest a group-bound self-similarity bias: peers that are similar to the target individual along certain dimensions, such as income, demographics, educational background, or behavior, are relatively more influential in inducing behavioral change (also [89]).

Role models constitute another essential component of a “natural” cultural learning environment. In the case of this bias, a model’s exhibited behavior is related to her success or status in order to heuristically assess the value of this trait. With their adoption decisions, role models also heighten the saliency of certain behaviors if they can draw on credibility or prestige assigned by local peers [72,90]. Studies show that “block leaders”, which belong to an individual’s proximal social network, provide a large effect on environmentally benign actions as to, for example, recycling or conservation, by exhibiting the corresponding behaviors [8]. Welsch and Kühling [81] report that people were more likely to install residential solar energy equipment if reference persons – neighbors, friends, or relatives as self-similar models – display this kind of consumption behavior. Furthermore, while being less powerful than direct communication with models in small group contexts, the media – and role models appearing therein – still can pave the ground for and foster the diffusion of behavior introduced to a group by more immediate means of communication.

Visibility, saliency, and assignability of the behaviors of role models or peers represent further aspects of ancient cultural environments. These are determinants of the effectiveness of biases in group-bound cultural transmission (also [91]). Accordingly, Babutsidze and Chai [92] find that peer behavior positively correlates with the adoption of visible climate change mitigation practices, but not with the adoption of non-visible mitigation behaviors. Similarly, promoting sustainable electricity consumption is difficult due to its non-visibility, both for the consumer herself and an observer [93–95]. Reductions in electricity consumption or building energy performance standards will, therefore, not easily become directly biased status-signaling lifestyle-choices. In contrast, the possession of solar systems is visible and suited to function as status-signaling items. Allcott and Rogers [12] report the use of home energy reports to make electricity consumption more visible and comparable by providing feedback on own consumption and comparisons with self-similar peer consumers. This abstract provision of information involving anonymous peers, however, only induced modest reductions in electricity consumption (also [11,36]). Furthermore, irrespective of the initial changes in overt “green” behavior, the decay of the “nudging” effect was significant. Agents did not acquire a preference for energy-saving behavior. In rather artificial learning environments, cultural transmission predicated on evolved cognitive biases turns out to be less effective in lastingly changing behavior.

A second scenario demonstrates the effect of an “enhanced green nudge” including role models on the diffusion of “green” preferences in a group of agents vis-à-vis a transitory change in behavior initiated by a conventional “green nudge” as described in Fig 1. The set of role models is constituted as follows: M2 and M3 are local peers whose high influence in cultural transmission emanates from direct interaction with the target individual and immediate visibility of behaviors ($\alpha_{2}=\alpha_{3}=\mathrm{.45}$). They represent a random draw from the peer group and mimic real face-to-face communication. M2 and M3 exhibit preference a or b and proxy for a “natural” learning environment with frequent encounters with self-similar models. Evolved biases are expected to be strong in such a setting. We also introduce an exclusively “green” model M1, whose weight in cultural transmission is $\alpha_{1}=\mathrm{.1}$. This model represents members of state and non-state institutions, non-local experts, or agents appearing on the media, such as politicians or scientists that are dedicated to promote “green” behavior. M1 captures more abstract learning channels at different societal levels with less weight in cultural transmission.

**Fig 2:**
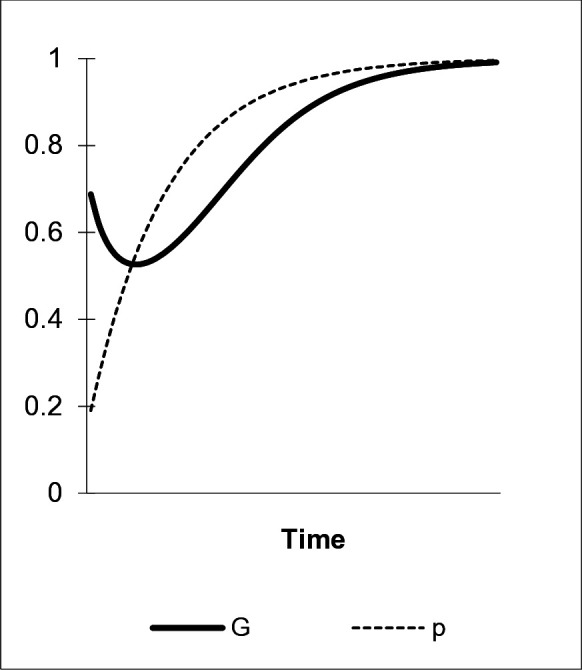
The influence of role models. $\alpha_{1}=\mathrm{.1}$, η=0, $\gamma_{ab}=0$, μ=.2, δ=0.

As shown in Fig 2, this relatively modest increase of M1’s influence as compared to the “fading scenario ($\alpha_{1}=0$), leads to the rapid spreading of the “green” preference a in the population, measured by p. Shifting some weight in model-based social learning from peers toward a “green” role model or media causes a slight increase in the adoption probability of a. Its frequency is raised in the population in the next time step including M2 and M3. Thereby, M1 initiates the transition toward a “green” consumption regime. However, the growing number of a types among the highly weighted, directly interacting peer models M2 and M3 is a prerequisite for this dissemination process to happen. Hence, group-bound “local” social learning in combination with a dedicated, more remote “green” model’s stimulus overcompensate the “fading effect” by inducing persistent preference change on the part of consumers. This translates into a level of overt “green” behavior at p=G. Due to agents’ socially malleable preferences, the decline of the overt “green” behavior is reversed. We also see that relative to the spreading of the “green” preference a the increase in the frequency of overt “green” behavior, G, is lagged. This indicates a temporary action-value-gap: while agents are endowed with preferences for environmentally benign behaviors, not all of them act according to these [92]. Consumers may need some time to adapt their lifestyles or to overcome habits and norms. This gap closes as more and more agents learn to act in line with their “true” acquired preference. We abstract from agents’ financial constraints in this context. Consequently, an “enhanced green nudge” incorporating a model bias moves the population toward an environmentally benign consumption regime, without changing monetary incentives or the option set itself.

An attempt to draw on the model bias in “nudging” are home energy reports that contain energy-saving achievements by an anonymous poster household [96]. However, since this abstract learning environment does not present “socially rich” contents, such as models from one’s peer group whose behaviors are visible and assignable, the bias does not induce persistent changes in preferences and the “nudge’s” effect fades. Fischer [93] finds that for changing a household’s electricity consumption in a lasting manner, conscious decisions in this direction are required that involve social learning along visible features of “green” models. In experimental settings, “nudging” strategies that involve concrete role models have been shown to be relativelymore effective in modifying consumption behavior [11]. Tangible role models agents identify and empathize with exert great influence on individuals’ behavior: marketing campaigns use prominent celebrities in advertisements to convince consumers to buy certain products [97]. This powerful force in social learning can also be harnessed to disseminate “green” behaviors [98]. In our model, this would be reflected by an increased weight of M1. Therefore, the model-based bias is a suited cognitive component of an “enhanced green nudge” meant to modify agents’ choice behaviors as well as underlying preferences. The bias’s impact in cultural transmission is, however, predicated on the characteristics of the learning environment it operates in. Evolved cognitive learning dispositions are most effective in environments resembling those from which they emanated during human phylogeny.

**Fig 3:**
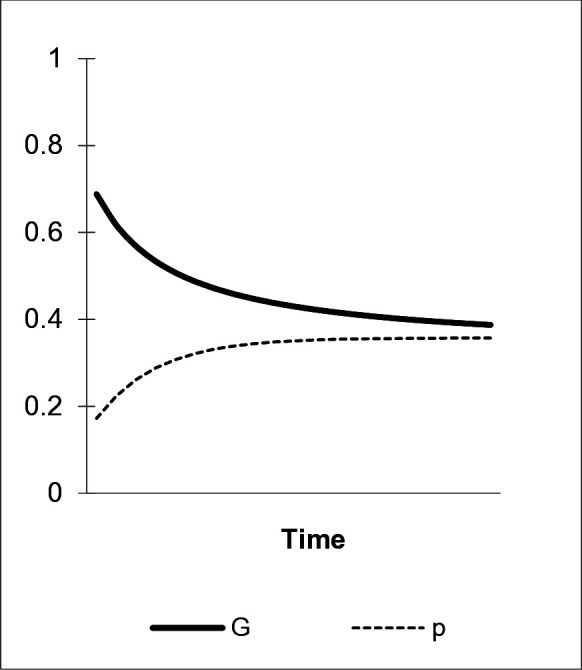
The hedonistic learning bias. $\alpha_{1}=\mathrm{.1}$, η=0, $\gamma_{ab}=\mathrm{.2}$, μ=.2, δ=0.

Given the parameter constellation in Fig 2 as a starting point for another scenario, we find that if agents are also subject to direct bias favoring b due to its status-signaling features or its superior hedonistic experiences in individual learning, the frequency of preference a in the population dwindles. In Fig 3, fading (μ=.2) together with a direct bias favoring preference $\begin{array}{c} b\;(\gamma_{ab}=\mathrm{.2}) \end{array}$ cause the level of overt “green” behavior, G, as well as the share of the “green” preference, a, measured by p, to reach significantly lower levels in the population ($\hat{p}=\hat{G}=\mathrm{.35}$). In this case, role model bias acting against hedonistic learning and decay is insufficient for the “green” preference’s extensive spreading in the population. Direct bias favoring a status-signaling or hedonistic alternative may threaten even established environmentally benign consumption regimes with high shares of preference a.

**Fig 4:**
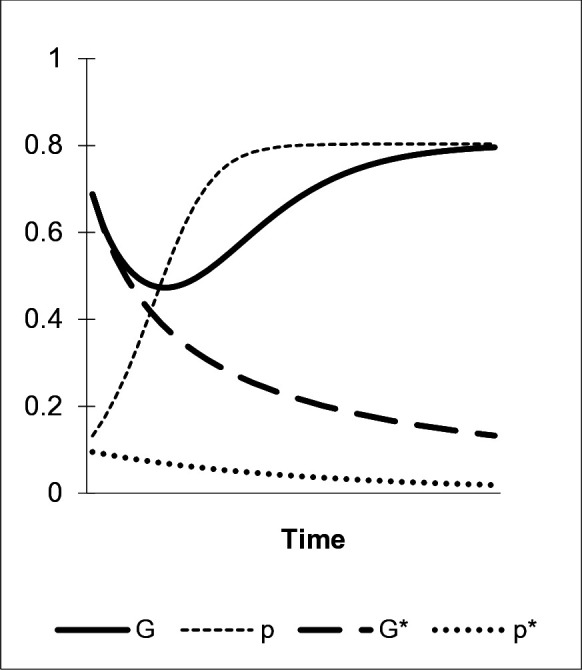
The conformity bias. $\alpha_{1}=0$, η=.2/.7, $\gamma_{ab}=\mathrm{.2}$, μ=.2, δ=0.

The next scenario accounts for the interactions of a “nudge’s” decay, direct bias favoring preference b, and conformity as a central component of an “enhanced green nudge”. Conformity is an influential force in group-bound learning and a typical characteristic of interactions in ancient as well as contemporary social learning environments. This evolved cognitive disposition draws on the observed frequency of stable behaviors and corresponding preferences in a reference group. Agents tend to adopt those traits exhibited by the majority. Conformity strongly affects the composition of individuals’ sets of preferences. As illustrated in Fig 4, a high level of conformity (η=.7) overcomes the simultaneous decay of G and p: the dedicated model M1 that exclusively exhibits the “green” preference a in combination with a-types among peers M2 and M3 constitute sets of role models where a is the more frequently exhibited trait. Since $\alpha_{1}=0$, M1’s influence exclusively manifests through the conformity bias. Conformity then spurs the dissemination of the “green” preference against the forces of fading and individual learning, as depicted by the development of p and G ($\hat{p}=\hat{G}=\mathrm{.8}$).

The involvement of local peers facilitates settings of strong conformity in cultural transmission that are pivotal to initiating this learning dynamic. Moreover, the increasing share of a among M2 and M3 accelerates the dissemination of the preference for the environmentally benign behavior. The rate of diffusion slows down again as the variance of a in the population decreases in the course of its proliferation. Finally, also a high level of the environmentally benign behavior A, measured by G, is reached. Thereby, the “green” preference, p, spreads following a typical S-shaped path of diffusion [69]. After an initial drop, the overt “green” behavior, G, exhibits a similar pattern of dissemination. Furthermore, while η=.7 implies strong conformity, also lower values of η induce significant levels of p: for example, as follows from Equations (6) and (7), for η=.4, we yield $\hat{p}=\mathrm{.47}$. As illustrated in Fig 4, weak conformity (η=.2) – potentially reflecting a more abstract learning environment in which majority behavior is not directly observed and assignable – does not suffice to induce “green” preference learning by consumers. Accordingly, the trajectories of G and p resulting from this parameter constellation, denoted by $G^{*}$ and $p^{*}$ in the figure, continuously decrease in time (for all η<.25, $\hat{p}=0$ holds).

Conformity as a facet of social learning-based preference acquisition in “natural” learning environments is an effective component of “enhanced green nudges”. Empirical evidence shows that the probability of agents to adopt “green” behaviors and underlying preferences is positively correlated with the number of adopters among their local peers [80,81]. Nolan et al. [99] show that the visible energy conservation behavior of the majority of an agent’s neighbors motivates to conserve more energy. In their study, this effect turned out to be the strongest amid other instruments. Moreover, conformity combines with norm-following behavior: agents are also normatively disposed to stick to the majority behavior [61]. Norms relating to environmentally benign behaviors frequently emerge within local reference groups where a majority of peers supports them and corresponding actions are easy to observe [92]. Sets of group-specific “green” norms are then stabilized by conformity as well as norm-following behavior and vary across regions’ idiosyncratic cultural environments. Both forces can, however, also stabilize environmentally harmful behavioral equilibria. Pioneer adopters as prestigious role models are then necessary to introduce environmentally benign behavior to the group to initiate a shift in local norms [100–102].

The following proposition highlights the potentially great effect of “enhanced green nudges” including a role model, norm-following, self-similarity, conformity, or direct bias in initiating enduring shifts in agents’ preferences:

**Proposition 2** *”Enhanced green nudges” that incorporate learning biases based on evolved cognitive dispositions effectively induce persistent changes in consumers’ preferences toward environmentally benign behaviors.*

Biases’ impact on cultural transmission is mediated by the learning environment: if its characteristics resemble those of the environment biases evolved from, the strength of learning dispositions is enhanced. An individual’s environment, therefore, affects which information enters the deliberate acquisition of preferences.

### 4.3. A default-based preference inducement effect

**Fig 5:**
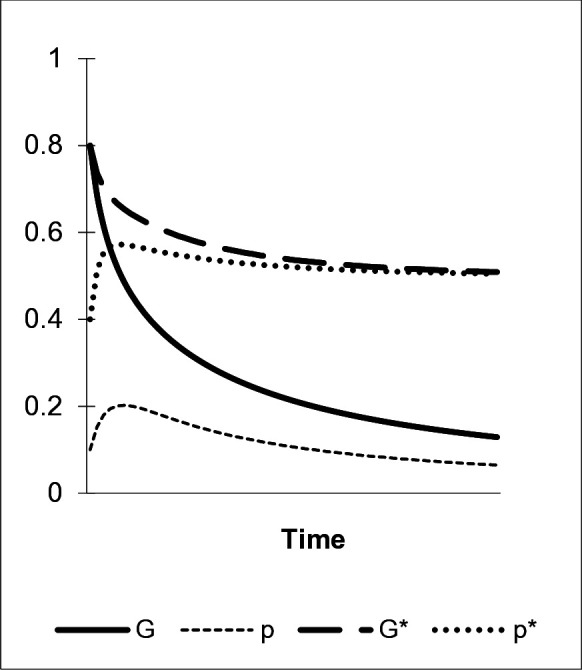
The inducement effect. $\alpha_{1}=0$, η=0, $\gamma_{ab}=\mathrm{.2}$, μ=.2, δ=.1/.4.

Our last scenarios consider “inducement effects” as a special feature of default-based “nudges”. Preset environmentally benign options established by this instrument cause some agents to shift preferences toward the “green” variant. Individuals develop a permanent preference for the default option due to a “status quo bias”. The latter combines an “endowment effect” with “loss aversion” that are both assumed to have deep roots in humans’ phylogenetic past. Because of that, this disposition qualifies as a component of an “enhanced green nudge”. To formally analyze the inducement effect’s potential impact on preference learning, consider a scenario as depicted by Fig 5. It shows how the inducement of “green” preferences through a default-based “nudge” can, *ceteris paribus*, significantly *raise the frequency of the environmentally benign behavior* A in the population, *G*, if its strength is sufficiently high.

Given the first parameter constellation in Fig 5, which includes a “nudge’s” fading process (μ=.2), a direct bias favoring b  ($\gamma_{ab}=\mathrm{.2}$), and a weak default-based inducement effect (δ=.1), the share of the “green” preference, a, as denoted by p, is – after an initial peak – continuously decreasing approaching zero in the long-run (with $\alpha_{1}=0$, η=0, p=0 and G=1 in the beginning). The same happens to the overt “green” behavior G. Following from these parameters and Equations (6) and (7) above, which set up our dynamic system of preference acquisition, the equilibria of G and p, are given by $\hat{G}=\frac{\mathrm{.2}(5\delta -1)}{\delta }$ and $\hat{p}=\frac{\delta G}{\mathrm{.2}+\delta G}$. With weak inducement G and p converge to zero ($\hat{G}=\hat{p}=0$), which holds true for all values of δ≤.2. However, for a stronger inducement effect (δ=.4), the shares of $p^{*}$ and $G^{*}$ converge toward an equilibrium at $\hat{G}=\hat{p}=\mathrm{.5}$. Consequently, strong default-based “nudges” counterbalance the effects of hedonistic learning and fading, stabilizing a significant share of the environmentally benign behavioral variant. This finding is consistent with empirical evidence on defaults that have been shown to implement high frequencies of “green” behavior in a population even if consumers’ were holding opposing attitudes (see the example of “Schönau” above).

**Fig 6:**
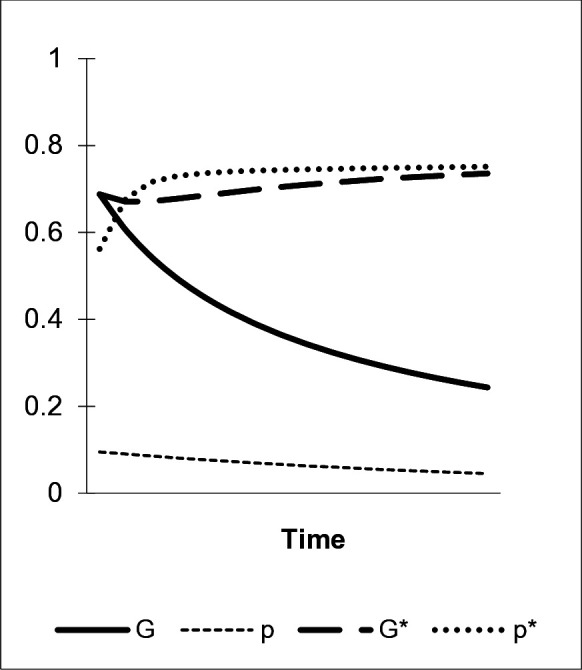
Conformity and inducement. $\alpha_{1}=0$, η=.2, $\gamma_{ab}=\mathrm{.2}$, μ=.2, δ=0/.6.

Preference inducement by preset options can serve as an auxiliary means of environmental policy to lead consumers out of a lock-in to environmentally harmful behaviors. The first parameter setting in Fig 6 shows that given fading processes (μ=.2) and hedonistic learning ($\gamma_{ab}=\mathrm{.2}$), conformity alone (η=.2) is insufficient to stabilize the share of preference a and the overt “green” behavior G among agents ($\hat{p}=\hat{G}=0$). When, however, combined with a strong default-based “inducement effect” (δ=.6) that gives rise to more a -types in sets of models, conformity shifts the levels of a and the environmentally benign behavior, G, as shown by the development of $p^{*}$ and $G^{*}$ ($\hat{G^{*}}=\hat{p^{*}}=\mathrm{.76}$). Lower values of δ still stabize p and G at significant levels given the parameters in Fig 6 (e.g., for δ=.1, $\hat{p}=\hat{G}=\mathrm{.29}$). Hence, especially “enhanced green nudges” that combine several evolved learning dispositions are effective in shifting preference acquisition dynamics. As to default-based nudges, Proposition 3 states:

**Proposition 3** *Default-based “nudges” induce preference learning, rest on evolved cognitive dispositions, and are well-suited components of “enhanced green nudges”.*

## 5. Conclusion

Most “green nudges” do not induce persistent change in consumers’ preferences, but merely trigger short-term behavioral adjustments that are subject to decay. In contrast, we showed that “enhanced green nudges” have the potential to effectively foster consumers’ acquisition of stable environmentally benign preferences. They incorporate powerful learning biases, such as direct bias, role model bias, self-similarity, norm-following, or conformity, that are based on evolved dispositions of human agents. To a great extent, these cognitive features determine which cultural information enters agents’ processes of preference acquisition. The effectiveness of “enhanced green nudges” is, however, mediated by the learning environment: if it shares key characteristics with the environment biases evolved from in humans’ phylogenetic past, these dispositions exert great influence in cultural transmission. Key features of these settings have been direct interaction and face-to-face communication in local small-group contexts as well as visibility, saliency, and assignability of peers’ behaviors. Since many human cognitive dispositions evolved in the context of bands, biases in social learning are powerful in environments characterized by features of “small group interactions”. When implementing “enhanced green nudges” as a political tool, a central task for politicians is to create learning environments that boost their effects.

Given these insights into “green nudging”, some implications for environmental policy can be derived. For example, a political initiative meant to promote “green” choice based on a role model and a self-similarity bias as components of an “enhanced green nudge” may comprise the following features: (1) the organization of direct communication between dedicated and acknowledged “green” experts and interested individual pioneer adopters, institutions, or firms about the environmental (and economic) characteristics of new “green” commodities or services. Potentially, this induces the acquisition of a “green” preference by first movers and the adoption of the environmentally benign novelty. Fairs, exhibitions, or competence centers could be (state-aided) frameworks for such an exchange of knowledge. (2) The early “green” adopters, in turn, introduce the environmentally benign behavior to their local group of self-similar peers by means of face-to-face contacts and direct communication, which are typical characteristics of an ancient learning environment. Due to past forward-looking behavior and corresponding success, these pioneers may have earned prestige within their group. As this is an essential trait for a model bias to take effect in cultural transmission, these key agents then spur the dissemination of the new “green” behavior. After having convinced a critical number of adopters in a rather small local community, this diffusion process – potentially backed by politics – is supported by conformity and may spillover to other groups.

Moreover, pecuniary incentives given by the government to promote the initial adoption of environmentally benign products or services increases their visibility in local groups (“eye-catching strategy”, see [103]). They also suggest a state-backed new consumption norm. Direct communication of (self-similar) peers triggered by this increased visibility spreads knowledge about the features of this novelty as well as the reasons for implementing it as a new behavioral norm. Individuals’ evolved psychological inclination to follow norms then disseminates the corresponding “green” behavior. This may happen even if the new behavior bears some additional costs: the “psychic force” emanating from the norm-following disposition would then dominate pecuniary motives. Again, if a critical mass of norm adopters is surpassed in the peer group, conformity accelerates the further diffusion of the “green” alternative. In addition, norms are defined in the context of societal debates. Politicians, the media, scientists, celebrities, environmental interest groups, or pioneer users are part of this norm creation process. “Green” norms can be made more salient by these prestigious models by engaging in these debates and by broadly integrating local societal groups. Individuals may, however, differ in their susceptibility to models’ particular influences. Political polarization, conspiracy theories, or distrust in science may impact on their subjective credibility or prestige. This should be considered when implementing “enhanced green nudges”.

Furthermore, a shift in a group’s norms toward environmentally benign behaviors opens up the possibility of “green” status races. “Enhanced green nudges” that incorporate directly biased cultural transmission of information on status signaling by means of environmentally benign consumption can induce corresponding status-related preferences [2,80]. Then, consumers’ desire to display “green” status to peers would also be an effective political instrument. Electric cars, for example, may be favored by a direct bias as “green” positional goods that indicate a progressive sustainable lifestyle [104]. Public support for this technology could then draw on this bias to speed up its diffusion. Thus, orchestrated sequences of “enhanced green nudges” would be implemented by policy to spread the “green” behavior.

The bulk of information humans as cultural beings process originates from (biased) learning mediated by characteristics of the environment. To a great extent, individuals’ preferences are formed on this informational basis. We suggested that “enhanced green nudges” are more effective in changing consumers’ preferences as compared to other “green nudges”: they draw on biases in cultural transmission predicated on cognitive dispositions with deep evolutionary roots in human phylogeny. In combination with suited learning environments, this class of “green nudges” represents a powerful political instrument to induce agents to adopt preferences for environmentally friendly commodities and services.
